# Efficacy of Calcium-Containing Eggshell Powder Supplementation on Urinary Fluoride and Fluorosis Symptoms in Women in the Ethiopian Rift Valley

**DOI:** 10.3390/nu13041052

**Published:** 2021-03-24

**Authors:** Demmelash Mulualem, Dejene Hailu, Masresha Tessema, Susan J. Whiting

**Affiliations:** 1 School of Human Nutrition and Food Science, Hawassa University, P.O. Box 5 Hawassa, Ethiopia; solymico27@gmail.com; 2 School of Public Health, Hawassa University, P.O. Box 5 Hawassa, Ethiopia; dejenkassa@yahoo.com; 3 Ethiopian Public Health Institute, P.O. Box 1242 Addis Ababa, Ethiopia; masresha88@gmail.com; 4 College of Pharmacy and Nutrition, University of Saskatchewan, Saskatoon, SK S7N 5E5, Canada

**Keywords:** calcium, fluoride, fluorosis, women, Ethiopia, Rift Valley, eggshell, dental

## Abstract

Dietary calcium binds Fluoride (F), thus preventing excess F absorption. We aimed to assess the efficacy of supplementing calcium-containing Eggshell Powder (ESP) on F absorption using urine F excretion and on fluorosis symptoms. In total, 82 women (41 Intervention Group, IG; 41 Control Group, CG) were recruited; overall, 39 in each group completed the trial. Morning spot urine was collected before (baseline, BL) and after (endline, EL) the intervention that was 6-months daily supplementation with 2.4 g ESP (providing ~1000 mg of calcium). Dental, skeletal, and non-skeletal fluorosis assessments was carried out at BL and, except for dental, at EL. Relative risk (RR) and linear generalized estimating equation were used to compare outcomes between groups. At BL, urinary F excretion in the IG and CG groups was similar, ~10 mg/L. At EL, urinary F excretion in IG women was six-fold lower (β = −6.1 (95% CI: −7.1, −5.1)) compared to CG. The risk of developing skeletal and non-skeletal fluorosis were significantly (*p* < 0.001) reduced in the intervention group. A significant reduction in urinary F excretion and reduction in many fluorosis symptoms were observed among women supplemented with calcium-containing ESP, thus providing evidence for using this dietary calcium source for mitigation of fluorosis. Clinical trials registration: NCT03355222.

## 1. Introduction

Worldwide, 50 million people suffer from fluorosis, which affects not only teeth, but also bones, joints, gut, and brain functions [1]. In Ethiopia, where defluoridation requires costly infrastructure, more than 14 million individuals, mainly in the Rift Valley, are affected by fluorosis [2,3,4,5]. Research suggests that the adverse effects of fluoride (F) can be reversed or lessened by providing sufficient food intake of protein, calcium (Ca), anti-oxidants, and vitamin D [6,7,8]. Of these, Ca is the most studied by ecological studies [8,9,10,11]. However, there have been no intervention studies of Ca to mitigate fluorosis at the community level in Ethiopia. 

Only a few studies have provided evidence of a reduction in F absorption with dietary Ca supplementation. A total of two animal studies [12,13] examined aspects of the mechanism and dose-responsiveness of Ca intake to reduce urinary F. A small human trial of 10 per group [14] found decreased urinary F excretion. Ca binds with F forming calcium-fluoride, which is insoluble in the gastrointestinal tract, preventing absorption (measured as a fall in urinary F) and therefore reducing the extent of F exposure. In this way it is postulated that the adverse effects of F are decreased and/or do not worsen with Ca intake.

We therefore hypothesized that supplementation of an age-old, sustainable and low cost source of Ca, i.e., eggshell, as a dietary Ca source [15] would mitigate the toxic effects of excess F intake in child-bearing women. High F exposure may be an added concern on women’s bone, dental, and overall health where there is low Ca intake such as in Ethiopia [16] and where water F is greater than the World Health Organization (WHO) limit of 1.5 mg/L [5,11,17]. Thus, the aim of this study was to test the efficacy of calcium-containing eggshell powder (ESP) supplementation, as a proof of concept, to reduce F absorption as measured by urinary F excretion (a primary outcome measure) and mitigation of fluorosis symptoms (secondary outcomes) in women living in a fluorosis-affected area. 

## 2. Materials and Methods

### 2.1. Study Design and Sample Size

This was a community-based Phase 2 clinical trial testing the efficacy of calcium-containing ESP supplementation compared to no supplementation. As a Phase 2 trial, our primary aim was to detect an effect of consuming calcium-containing ESP by mothers on F excretion. Mehta and Shah [14], using 10 subjects per group, found that Ca supplementation significantly reduced urinary F excretion. As we also evaluated fluorosis symptoms, the sample size estimation to detect the effect of intervention on fluorosis was done using EpiInfo Version 7.0.8.3 software (The United States Centers for Disease Control and Prevention, Atlanta, GA, USA) with double population proportion formula by considering the percentage outcome in exposed group (P1 = 90%, percent of pain and rigidity in the joints before intervention which is a major skeletal and non-skeletal fluorosis symptoms) and unexposed group (P2 = 60%, percentage recovery during intervention) from a published study [6]. A minimum sample size of 76 women (38 for each arm) was estimated to detect the effect of intervention on fluorosis symptoms using power (1-β) of 80% at 95% two-sided confidence level for a one-to-one exposed and unexposed ratio. Then we increased the sample size to 41 from each kebele to account for variability in symptom prevalence and loss to follow up. 

### 2.2. Participant Selection 

**Figure 1:**
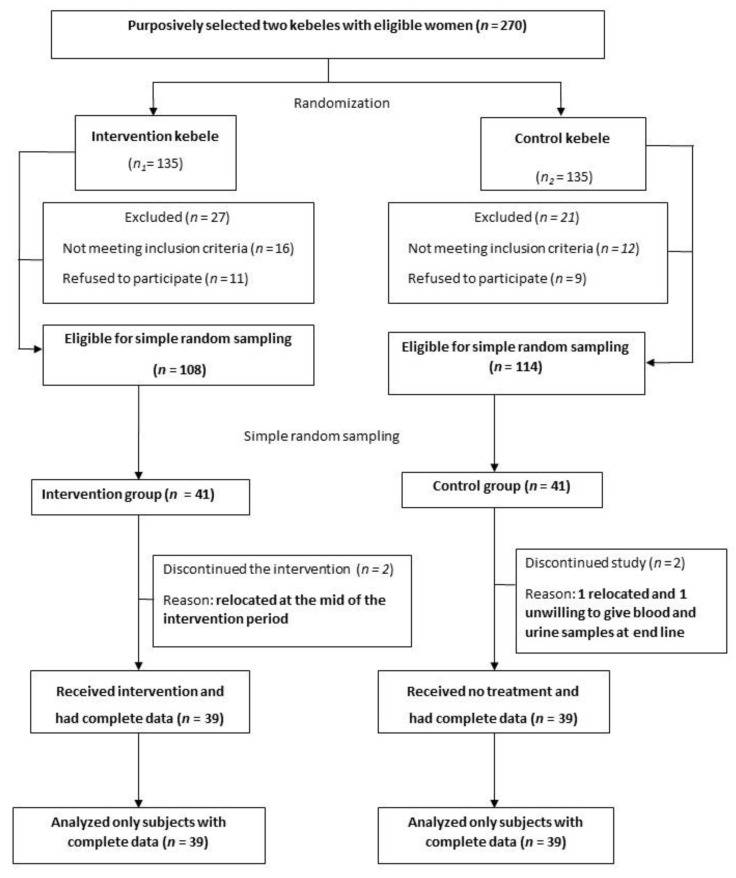
Flow diagram of the progress through the phases of the clinical trial.

This trial randomized two purposely selected clusters which were distant rural kebeles (villages) of Halaba district having fluorosis, located in the main Rift Valley of Southern Ethiopia. The participant inclusion criteria were being a biological mother of a child aged 6 to 18 months and permanent residents in the study area. Mothers who were taking drugs for any type of illness, had any apparent signs and symptoms of diseases such as fever and cough at the time of enrollment and those enrolled in any other health and nutritional intervention program were excluded from participation (Figure 1). A list of mothers was obtained from the family folder in each kebele’s health post to conduct a house to house registration to identify eligible households. At this time, exclusive codes were given for each eligible household. Thereafter, as the number of participants required for the study was smaller than eligible women in each cluster, within the respective selected kebeles, the sample was randomly selected from the list of eligible mothers (sampling frame) using the IBM SPSS version 25 statistical software (Chicago, IL, USA, 2017). The overall progress of subjects through the phases of the clinical trial is depicted in Figure 1.

### 2.3. Recruitment Period and Follow Up

Participant recruitment took place during the baseline survey (10 January 2018 to 21 February 2018) based on the eligibility criteria. The implementation of the intervention was conducted for six consecutive months (from 1 April 2018 to 30 September 2018) with weekly follow up and supervision. During the follow-up and supervision, assessment was made regarding to the ESP consumption including: likes and dislikes of the ESP, asking whether the women has been taking the recommended amount, asking whether the ESP was shared for the family, checking if the remaining amount was enough until new provision of ESP made and asking about any barrier to ESP consumption. In addition, observation was made while the women were consuming the ESP during the follow up and supervision period. The trial ended when the study subjects had received the intervention for the intended period of time (6 months).

### 2.4. Randomization and Masking

The two kebeles were purposively selected for feasibility reasons for the trial on the basis of having high F levels in the drinking water sources [11]. A lottery method (flipping of a coin) was done to assign the two kebeles (clusters) either to the intervention or control arm. The physiotherapist who did the skeletal and non-skeletal fluorosis assessment, the laboratory technologists who collected or analyzed blood and urine specimens, and the data collectors who did the interviews were kept uninformed with respect to the intervention allocation. All biochemical samples could only be identified with exclusive codes assigned for each participant. 

### 2.5. Intervention 

The intervention (exposure) was daily supplementation of 2.4 g of calcium-containing ESP for six months that would provide approximately 1000 mg Ca per day. The powder was provided to women in monthly doses. The women were provided with plastic caps (from plastic soft drink bottles) which are common measuring tools. Women were instructed to take a half cap three times daily where each half cap provided ≈800 mg ESP (equivalent to 320 mg Ca). Women were instructed to take each dose at three different times over the day (i.e., not all at once). For this trial, eggshells were originally planned to be available to families through provision of chickens; however, to maintain a consistent supply and quality, eggshells were purchased from local restaurants and processed as described below. When families later began accumulating eggshells from their donated chickens, we purchased eggshells from women in the community. 

White eggshells were washed, boiled for 10 min, dried, and then crushed before being processed into a fine powder using High–speed Multi-functional Grinder (^®^RIRIHONG, RRH–A1000, Shanghai Yuan wo Industrial and Trade Co. Ltd. Manufacture: Hongtaiyang Electrical and Mechanical Services Co. Ltd., Yongkang City, Zhejiang Province, China). After sieving, the ESP was packed in 100 mL screw-capped polyethylene bottle before it was distributed to subjects in the intervention group. Then, two local coordinators, four health extension workers and eight health development agents were organized to distribute packaged ESP in the intervention kebele and conduct the monitoring and follow up activities. There was no placebo distributed in the control kebele. At every fourth week time point, new ESP bottles were distributed and old bottles, which were used as one of the measures of adherence that we are reporting separately, were returned. Participants were instructed to add the recommended amount of ESP to cooked foods or drinks ready to be eaten or drunk only by them. The health development agents motivated study participants in their one-to-five networking sessions and provided weekly reports to the local coordinators and health extension workers. So, when necessary, corrective measures and encouragement to the appropriate consumption of the ESP could be done during each weekly follow-up period.

### 2.6. Data Collection and Measurements

Our outcome measures were decreased urinary F excretion (primary outcome) which is an indirect measure of F absorption, and mitigation of skeletal and non-skeletal fluorosis symptoms (secondary outcomes). Data on socio-demographic and economic characteristics were collected using a semi-structured questionnaire that was in the language of the district (*Hallabena*). The interview was conducted face-to-face with the subjects by trained data collectors. The BL data collection (including biological data and fluorosis assessment) was conducted from January to February 2018. At EL (October 2018) the second-round data collection and measurements were done. 

#### 2.6.1. Diet Diversity 

At BL, we collected data on specific foods consumed by women in the previous 24 h using a structured questionnaire in order to determine the diet diversity score. The recall was repeated on 10% of women to determine if these recalls were similar when done on different days of the week. In order to determine an individual diet diversity score, foods were placed into each of 10 food groups. Women who consumed five or more food groups in that day were considered to have fulfilled the minimum diet diversity [18].

#### 2.6.2. Dietary Calcium Intake

At BL, to estimate the usual dietary Ca intake of women, daily and weekly frequencies of consumption of specific calcium-rich foods common in this district (milk, yogurt, millet, fruits, kale, other vegetables, and moringa) were assessed using a structured food frequency questionnaire. Portion size estimation was made using bowls and plates available in the household of each woman to help them visualize the amount of each food consumed. These frequencies were converted to daily dietary Ca intakes using published values for Ca content [19]. 

#### 2.6.3. Urine Fluoride

At BL and EL morning spot urine specimens were collected in a screw capped plastic bottle from the intervention and control women. Everyone who provided a urine specimen gave at least 30 mL. The urine spot collection was done in the morning between 8:00 and 10:00 AM, which was several hours after the women had gotten up for the day. The samples were labeled with a unique code and kept in an ice box until they were brought to the laboratory for F, creatinine, and Ca concentration analysis. The ice box was cooled with ice packs which were changed at 12 h intervals to prevent the samples from decomposition. The urine specimen was stored at −20 °C for 3–6 weeks in a refrigerator at EPHI’s laboratory until it was analyzed.

The F concentration in urine samples were analyzed using PerfectIONTM combination F ion selective electrode (Mettler Toledo, Gießen, Germany) coupled with bench top dual channel ion-meter (Jenway, model 3345, Cole-Palmer, Staffordshire, UK), using facilities at EPHI. Total ionic strength adjustment buffer (TISAB) solution: 58 g sodium chloride, 2 g EDTA, 7 g tri-sodium acetate, 57 mL glacial acetic acid, and 500 mL distilled water were dissolved. The pH of the solution was adjusted to 5.0 to 5.5 by using 5 M NaOH solution and the volume was adjusted to 1000 mL with distilled water. Stock standard solutions (1000 ppm) were prepared by dissolving 2.21 g of sodium fluoride (NaF) with 1 L of distilled water. Then, 100, 10, 5, 1, and 0.1 ppm working standards were prepared from the stock standard by using consecutive dilutions. During analysis, 5 mL of buffer solution pipetted into a measuring cell followed by 5 mL of the urine sample. An equal amount of buffer solution was also added for working standards. The electrode was calibrated by working standards starting from most diluted to concentrated standard. The concentration of the solutions was directly measured in ppm by using a fluoride ion-selective electrode, as described elsewhere [10,14].

The calcium and creatinine (CR) concentrations in urine samples obtained on a Roche/Hitachi cobas^®^ c 501 analyzer (Roche Diagnostics International AG, Rotkreuz, Switzerland) using the Roche Calcium Gen.2 and Creatinine plus ver.2 reagents, respectively. These concentration values were compared with those determined using the Roche Calcium reagent on a Roche/Hitachi MODULAR P analyzer for urine calcium and using the corresponding reagent on a Roche/Hitachi 917 analyzer for urine creatinine. The urine sample was analyzed in duplicate at the EPHI. All the analysis (F, Ca, and CR) were analyzed against blank samples, and along with standards for quality control. Moreover, Certified Reference Materials were analyzed along the samples to check the accuracy and sensitivity of the method. 

#### 2.6.4. Dental Fluorosis

At BL, the women were assessed for dental fluorosis using Dean’s index [20] which classifies fluorosis on a scale of 0 to 4 as: Class 0, No Fluorosis; Class 1, Very Mild Fluorosis (opaque white areas irregularly covering 25% of the tooth surface); Class 2, Mild Fluorosis (white areas covering 25–50% of the tooth surface); Class 3, Moderate Fluorosis (all surfaces affected, with some brown spots and marked wear on surfaces subject to attrition); and Class 4: Severe Fluorosis (widespread brown stains and pitting). The average score of the two most severely affected teeth was used to derive this classification. The women were instructed to thoroughly clean their teeth prior to the dental fluorosis examination. The dental assessment was done by an experienced dentist (MD, DDS) who was hired for this purpose. 

#### 2.6.5. Skeletal and Non-Skeletal Fluorosis 

Skeletal and non-skeletal fluorosis level were determined at BL and EL using clinical symptoms and physical exercises as developed by Susheela et al. [21] and Shashi et al. [22] and as described by Kebede et al. [11]. Individuals who could not adequately perform the physical exercises were categorized as having skeletal fluorosis. The assessment was carried out by an experienced physiotherapist (BSc, MSc degree) who was hired to perform the physical examination. All study women were asked to perform three physical exercises which indicate skeletal fluorosis: (i) bending the body and touching floor or toe, (ii) touching chest with chin, and (iii) folding arms to touch back of head. The physiotherapist showed each mother how to perform the exercise. The same physiotherapist assessed the women for the presence or absence of 13 symptoms of skeletal and non-skeletal fluorosis as described by Tandon et al. [1]. 

### 2.7. Dropouts

During the middle of the intervention, two women from the treatment group and one woman from the control group moved to a new location outside the study kebeles. At the end of the intervention, one woman from the control group was unwilling to provide urine and blood specimens and thus excluded from the study (Figure 1). Efforts were made to minimize study participants’ attrition in both groups during the intervention period by close follow-up and providing incentives, such as soap and iodized salt. 

### 2.8. Statistical Methods

All data were double entered into EpiData version 3.1 (Odense, Denmark) and then exported into IBM SPSS version 25 (Chicago, IL, USA) for analyses. Continuous data were checked for normality using Shapiro–Wilk Test of Normality. The Chi-square (X2) test was used to compare baseline characteristics between the intervention and control groups. Relative risk (risk ratio) was computed to compare the dichotomized outcome variables (skeletal and non-skeletal fluorosis symptoms) at BL and EL. Paired samples *t*-test was used, for each group, to compute the ‘within’ mean differences of baseline and endline urine F, Ca, and CR levels. The generalized estimating equation (GEE) which is an extension of the generalized linear model (GLM) was used to investigate the relationship of repeated measurements (the pre and post intervention continuous data on urine F, Ca and CR levels). GEE helps to correct within subject correlation and uses quasi-likelihood estimation which allows for over dispersion [23]. The probability distribution in GEE was a quasi-likelihood function and the working correlation matrix structure was independent. GEE does not require the homogeneity of variance to be satisfied. In this study, urine F was normally distributed, but urine Ca and CR were not normally distributed. Therefore, a linear generalized estimating equation was used to allow the repeated measures of continuous variables and non-normally distributed data (urine Ca and CR levels). Mother age and parity, family size (number of children, parents and relatives living together in the household), year of residence in the area, diet diversity, and dietary Ca intake were included in the multivariate analysis of GEE model. Then, the multivariate linear regression analysis was carried out and beta coefficient at 95% confidence interval (CI) reported. 

### 2.9. Ethics 

Ethics approvals were obtained from Institutional Review Board of Hawassa University College of Medicine and Health Science, Ethiopia (IRB/019/10, on 12 December 2017), and University of Saskatchewan Biomedical Research Ethics, Saskatoon, Canada (BIO-REB-17-150 on 25 July 2017). Letters of support were also obtained from Regional Health Office, Zonal Health Desk and District Health Offices. Confidentiality of personal information has been kept. After the purpose and methods of the study were fully explained, and their right to refuse was explained, informed verbal and written consent were obtained from all study participants prior to their participation in the study.

## 3. Results

### 3.1. Socio-Demographic, Economic and Nutritional Characteristics

At BL, the mean (±SD) age of women in completed years was 29.6 ± 3.8 years. There was no significant difference (t = −0.91, DF = 76, *p* = 0. 368) in the distribution of age between the two arms. Most of the women (93.6%) were married and more than half of them were illiterate (56.4%) and had no job outside their household (53.8%). The main source of income for most (88.5%) of the households was agriculture and the majority (83.3%) of them earned an average of less than 1000 Ethiopian birr per month, equivalent to approximately $25 USD (Table 1). The mothers’ average number of live-born children was approximately four. The average family size (parents, children, and relatives living together) was approximately 7 ± 2, in which at least two of them were children under five years old. Overall, the groups were comparable at baseline and the differences were small (Table 1). 

About two-thirds of the women did not fulfill the minimum daily diet diversity score (consuming at least from 5 food groups out of the 10 food groups). The mean (SD) dietary diversity score (DDS) of women based on ten food groups was approximately 5 ± 2. The average dietary Ca intake was 403 ± 124 mg, and the independent two samples *t*-test showed a small and non-significant mean differences in the DDS (t = 0.74 (DF = 76), *p* = 0.459) and in the mean difference of dietary Ca intake (t = 0.65 (DF = 76), *p* = 0.516) between the intervention and control groups at BL (Table 1). 

### 3.2. Baseline Dental Fluorosis

The proportion of severe dental fluorosis among all women included in this trial was 10.3%; and the proportion of women with moderate dental fluorosis was 29.5% (30.8% in the intervention and 28.2% in the control groups). Overall, more than half, 57.7% (64.1% in the intervention and 51.3% in the control groups) of the study women had very mild to severe dental fluorosis level. There was no statistically significant difference in the proportion of dental fluorosis categories between the treatment and control groups during the baseline assessment (*p* > 0.05) (Table 2). As dental fluorosis is assumed to be an irreversible process, the dental examination was not repeated after the end of the trial.

### 3.3. Urinary Fluoride, Calcium and Creatinine 

At BL, the independent two samples *t*-test showed that the average urinary F (~10 mg/L) excretion by the women between the intervention and control groups was similar (t = 0.11 (DF = 76, 95% CI: −1.31, 1.47)). After the calcium-containing ESP supplementation, there was more than 50% (Table 3) significant reduction in the urinary F excretion of women in the intervention group compared with their baseline measurements using paired samples *t*-test; (t = 5.7 (DF = 76, 95% CI: 4.77, 6.49)). At endline, women in the intervention group had about six-fold lower urinary F excretion (β = −6.1 (95% CI: −7.1, −5.1)) compared to women in the control group (Table 3). Urinary CR excretion was used as a measure of the spot urine collection. As shown in Table 3, all four collections of CR showed similar average concentrations of CR. There was no significant difference between the intervention and control groups in CR concentration at BL (*p* = 0.785) and EL (*p* = 0.571). In addition, there was no significant difference in urinary Ca excretion among the groups prior (*p* = 0.945) and after (*p* = 0.247) the supplementation of calcium-containing ESP (Table 3).

### 3.4. Skeletal Fluorosis Using Physical Exercise Testing 

At baseline, the overall proportion of physical signs of skeletal fluorosis among women in this trial was 41.3% (43.6% in treatment and 38.5% in the control group). The two groups were similar (*p* > 0.05) in all skeletal fluorosis exercise testing (Table 4). After the six months supplementation of calcium-containing ESP, the overall proportion of physical signs of skeletal fluorosis significantly decreased from 43.6 to 17.9% in the treatment group (*p* < 0.001), while the control group did not change. The risk of developing skeletal fluorosis tested using the ability to bend body and touch floor or toe (RR = 0.21 (95% CI: 0.07, 0.69)), and stretch and fold arms to touch back of head (RR = 0.18 (95% CI: 0.04, 0.77)) were significantly reduced in the intervention group by 79 and 82%, respectively, compared with the control (Table 4).

### 3.5. Pain and Muscular Symptoms of Non-Skeletal Fluorosis

At baseline, all pain and muscular symptoms of non-skeletal fluorosis were similar (*p* > 0.05) among the intervention and control group (Table 5). The majority of the women had lower back pain (71.8, 76.9 in the intervention group, and 66.7% in the control group) and tingling sensation in the hands and feet (70.5, 69.2 in the intervention group, and 71.8% in the control group). About two-third of them had also leg pain (64.1, 69.2 in the intervention group, and 59% in the control group) and muscle weakness (62.8, 64.1 in the intervention group, and 61.5% in the control group), which were not statistically different between the two groups (*p* > 0.05) (Table 5). A majority of the women in IG reported mitigation of pain and muscular symptoms of non-skeletal fluorosis ranging from lowest RR = 0.17 (95% CI: 0.05, 0.52) to highest RR = 0.59 (95% CI: 0.39, 0.88) after the calcium-containing ESP supplementation than in CG (Table 5).

### 3.6. Gastrointestinal and Urinary Symptoms of Non-Skeletal Fluorosis

At baseline, the two groups were comparable (*p* > 0.05) in all gastrointestinal and urinary symptoms of non-skeletal fluorosis (Table 6). The majority of the women complained of constipation (79.5% in the intervention group and 87.2% in the control group). Just half of the women had nausea (53.8% in the intervention group and 48.7% in the control group). Only very few women reported gastrointestinal or urinary symptom of non-skeletal fluorosis. After the six months supplementation of calcium-containing ESP, the risk of developing gastrointestinal symptoms such as loss of appetite and nausea, but not constipation or bloating were significantly reduced in women in the IG compared with those in the CG. The risk of developing bladder-related symptoms such as polyuria and polydipsia was not different from baseline after six months of calcium-containing ESP supplementation and not different from that in women who received no ESP (Table 6).

## 4. Discussion

In this area, water F measured in different water sources as approximately 5 mg/L, which is close to a previous estimate [11]. Additionally, F was detected in all locally grown foods (unpublished). At baseline, we found urinary F levels to be high, reflecting the F exposure of those living in this district of the Rift Valley. The F excretion by the women, averaging 10.2 mg/L in urine, implies that women were exposed to about 20 mg per day, assuming urine excretion to be at least 2 L per day. Our data were twice the Upper Level of 10 mg per day set by the Institute of Medicine [24]. The primary preferred option for fluorosis is to find a supply of safe drinking water using de-fluoridation [2,25,26]. However, in these communities there was no defluoridation of the high fluoride-containing water. As well, there were no fluorosis prevention strategies being attempted. In this area, rainwater, a potential source of low F drinking and cooking water, was not used for these purposes as it was scarce and due to fearing of dust contamination from the roof of the house. 

Currently in Ethiopia, eggs are promoted as an excellent animal source food [27,28,29]. Our previous research had shown that the use of eggshell powder by young children in Ethiopia was acceptable [28]. However, we had not linked its use to fluorosis. Therefore, this study assessed the effects of ESP supplementation as a source of Ca on fluorosis symptoms and body F handling in women using urinary F as a biomarker. Eggshells have been used to provide Ca in other settings [15,30,31,32,33,34]. They contain predominantly Ca carbonate and trace amounts of other micro-minerals such as iron, zinc, magnesium, and copper [30,34]. Chicken eggshells are reported to contain 2.07 ± 0.18 g of Ca and studies show that this source of Ca was equivalent to other types of Ca supplement sources [32]. In addition, adding eggshell to foods or beverages did not produce important changes in flavor and texture. Similar Ca absorption was found comparing diets fed to rats containing chicken eggshell powder to one with a calcium carbonate, both with equal Ca content [35]. Those authors, therefore, recommended chicken eggshell as a bioavailable Ca dietary supplement, as others have done [15].

At EL, the urinary F excretion was significantly decreased among women supplemented with ESP compared with the control group. This is in line with a clinical study conducted in India to evaluate the clinical reversal of dental fluorosis in adolescents (age 8 to 17 years) with various combinations of Ca, vitamin D3, and ascorbic acid. That study also measured serum and spot urine F levels, and no change in clinical grades of dental fluorosis was noted, however, a significant reduction in serum and urine F levels was observed among subjects either given daily Ca (250 mg) with once-weekly vitamin D supplements (60,000 IU) for three months [14]

That Ca reduces F absorption was studied in two animal studies. One study examined different doses of Ca (provided as calcium carbonate) that were administered to rabbits. Significant decreases in F excretion were observed, in a dose-response manner. The higher doses of 17.2, 21.4, and 50 mg Ca/kg body weight were equivalent to 1200, 1500, and 3500 mg of Ca in a 70 kg human. The authors predicted doses of 1200–3500 mg of Ca would be required in humans to mitigate the adverse effects of fluorosis [12]. In a second animal study [13], total daily F excretion, both urine and fecal, by rats given additional Ca and magnesium was monitored. This study supported the hypothesis that gastrointestinal binding of F ions by Ca occurred, as fecal F excretion increased in concert with a decrease in urinary excretion.

All the four urine collections showed similar average concentrations of CR which indicated that spot urines were collected in each group in a similar manner at BL and EL. A large but non-significant increase in urinary Ca excretion was observed among intervention women after supplementation with calcium-containing ESP. As these women had a low mean intake of dietary Ca (Table 1) of approximately 400 mg/day consistent with other women in Ethiopia [16], the additional Ca intake from ESP supplementation might contribute to fulfill the Recommended Daily Allowance (RDA) for many of the subjects receiving it. There was no evidence of hypercalciuria.

A significant mitigation of skeletal and non-skeletal fluorosis symptoms was observed among women who received additional Ca from ESP over 6 months in the present study. Women and children in this part of Ethiopia had been reported as having high prevalence of fluorosis symptoms [5,11] and low intake of dietary Ca [16]. These symptoms of pain and poor mobility are also true of osteomalacia due to low calcium and low vitamin D [36]. A study of women in southern Ethiopia found a high prevalence (84.2%) of insufficiency based on serum 25-hydroxyvitamin D levels <50 nmol/L; in total, 14.3% of women were deficient (<30 nmol/L) [37]. These data indicate a high probability of vitamin D inadequacy. To our knowledge, there are no data on parathyroid hormone levels of Ethiopian women. 

Traditional sources of Ca such as dairy and green leafy vegetables are not widely available. Experimental and clinical studies conducted to date have shown that eggshell powder is a viable source of calcium including remediation of osteoporosis [38]. Therefore, the current study provides a proof of concept demonstrating mitigation of fluorosis symptoms and reduction in F absorption among women supplemented with calcium-containing ESP for at least six months. 

The limitations of this study include lack of access to X-ray confirmation of skeletal fluorosis and thus, we used several non-specific clinical symptoms and physical exercise tests to assess skeletal and non-skeletal fluorosis. The effect of Ca on body F load was measured using urinary F excretion biomarker which is an indirect measure of F absorption. We did not take into account that in the situation of high F, calcium absorption may be reduced. This suggests that more information on dose response is needed, as only one study of rabbits [12] showed that higher doses of calcium binds more F. Dietary Ca intake of women was estimated from frequencies of calcium-rich food consumption which might not have accurately estimated the usual Ca intake of women beyond that month. As the trial was conducted at the community level, due to fear of information contamination, the randomization was done only at the kebele level. In addition, there was no placebo treatment for the women in the control group.

## 5. Conclusions

Mitigation of many of the symptoms related to skeletal and non-skeletal fluorosis and reduction in urinary F excretion were observed among women supplemented with calcium-containing ESP in a fluorosis prone area of Ethiopia. Therefore, this study provides a proof of concept on using a traditional source of dietary Ca, eggshell, for mitigation of fluorosis in an area where there is endemic fluorosis and low intake of dietary Ca. The potential feasibility, sustainability and safety of using home prepared crushed eggshells in areas where fluorosis is endemic need to be studied.

## Figures and Tables

**Table 1:** Baseline socio-demographic, economic and nutritional characteristics of study participants, Halaba district, southern Ethiopian Rift Valley, 2018.

Characteristics		Intervention Group (*n* = 39)	Control Group (*n* = 39)	*p*-Value ^¥^
**Socio-Demographic and Economic**				
Age (years)	Mean (SD)	29.9 (3.9)	30.5 (3.8)	0.368
		*N* (%)	*N* (%)	
Educational status	Illiterate	24 (61.5)	20 (51.3)	0.361
	Literate	15 (38.5)	19 (48.7)	
Main occupation	No outside job	22 (56.4)	20 (51.3)	0.650
	Outside job	17 (43.6)	19 (48.7)	
Main source of income	Agricultural	35 (89.7)	34 (87.2)	0.723
	Non-agricultural	4 (10.3)	5 (12.8)	
Average monthly income	<1000 ETB	31 (79.5)	34 (87.2)	0.362
	≥1000 ETB	8 (20.5)	5 (12.8)	
Parity	≤4 live births	21 (53.8)	20 (51.3)	0.821
	>4 live births	18 (46.2)	19 (48.7)	
Family size	≤5 members	10 (25.6)	13 (33.3)	0.456
	>5 members	29 (74.4)	26 (66.7)	
Residency	<10 years	12 (30.8)	9 (23.1)	0.444
	≥10 years	27 (69.2)	30 (76.9)	
**Nutritional**				
Diet diversity score	Mean (SD)	5.1 (1.99)	4.7 (1.96)	0.459
Dietary calcium intake	Mean (SD)	394 (120) *N* (%) 22 (56.4)	412 (129) *N* (%) 21 (53.8)	0.516
		
Average dietary calcium intake	≤400 mg/day			0.820
	>400 mg/day	17 (43.6)	18 (46.2)	
Dietary diversity score	≤5 food groups	24 (61.5)	27 (69.2)	0.475
	>5 food groups	15 (38.5)	12 (30.8)	

ETB = Ethiopian Birr; IG = Intervention Group; CG = Control Group. ^¥^ For values with mean (SD) comparison was done using independent samples *t* test; for prevalence values, comparison was done using Chi-square test.

**Table 2:** Comparison of prevalence of dental fluorosis between the intervention and control.

Dental Fluorosis Category ^¥^	IG (*n* = 39)	CG (*n* = 39)	Total (*n* = 78)	*p*-Value ^#^
	*N* (%)	*N* (%)	*N* (%)	
Normal	11 (28.2)	14 (35.9)	25 (32.1)	0.628
Questionable	3 (7.7)	5 (12.8)	8 (10.3)	0.712
Very mild	4 (10.3)	3 (7.7)	7 (9.0)	0.999
Mild	5 (12.8)	2 (5.1)	7 (9.0)	0.431
Moderate	12 (30.8)	11 (28.2)	23 (29.5)	0.999
Severe	4 (10.3)	4 (10.3)	8 (10.3)	0.999

IG = Intervention Group; CG = Control Group; ^¥^ Assessment was based on criteria of Dean’s index (Dean, 1934). ^#^ *p*-values were generated by chi-square test using OpenEpi Version 3.03.

**Table 3:** Urinary excretion of fluoride (F), calcium (Ca), and creatinine (Cr) of women in the intervention and control groups in Halaba district, Ethiopian Rift Valley, 2018.

Measure	Baseline	Endline	Within Mean Difference (95%CI) ^†^	Beta Coefficient (95%CI) ^¥^	*p*-Value ^†^
	Mean (95%CI) (*n* = 39)	Mean (95%CI) (*n* = 39)			
**F (mg/L)**					
**IG**	10.3 (9.2, 11.3)	4.6 (4.1, 5.2)	5.7 (4.77, 6.49) ^#^	− 6.1 (−7.1, −5.1)	<0.001
**CG**	10.4 (9.4, 11.3)	10.8 (9.9, 11.7)	−0.5 (−0.96, 0.05)	1	
**Ca (mmol/L)**					
**IG**	0.52 (0.24, 0.80)	0.82 (0.50, 1.13)	−0.3 (−0.74, 0.15)	0.3 (−0.27, 0.78)	0.346
**CG**	0.53 (0.34, 0.73)	0.57 (0.30, 0.85)	−0.04 (−0.38, 0.29)	1	
**CR (mmol/L)**					
**IG**	4.8 (3.8, 5.7)	5.0 (4.2, 5.7)	−0.2 (−1.2, 0.9)	0.5 (−1.1, 2.2)	0.542
**CG**	5.0 (4.0, 5.9)	4.6 (3.7, 5.6)	0.4 (−1.0, 1.7)	1	

^†^ The ‘within’ mean difference of endline from baseline computed using paired samples *t*-test. ^#^ Statistically significant difference observed at *p* < 0.05; ^¥^ Beta coefficient at 95% Wald confidence interval and *p*-value analyzed using GEE linear model. ¥ Urinary fluoride, calcium and creatinine (CR) were adjusted for age, parity, family size, residency, diet diversity and dietary Ca intake. IG = Intervention Group; CG = Control Group.

**Table 4:** Skeletal fluorosis (SF) exercise testing in a 6-month eggshell powder intervention between intervention and control groups in Halaba district, Ethiopian Rift Valley, 2018.

		Skeletal Fluorosis Exercise Testing ^§^, N (%)					
	Group	Bend Body and Touch Floor or Toe		Stretch and Fold Arms to Touch Back of Head		Touch Chest with Chin	
		SF	No SF	SF	No SF	SF	No SF
Baseline	IG (*n* = 39)	15 (38.5)	24 (61.5)	17 (43.6)	22 (56.4)	19 (48.7)	20 (51.3)
	GC (*n* = 39)	13 (33.3)	26 (66.7)	15 (43.6)	24 (56.4)	17 (43.6)	22 (56.4)
	RR (95%CI)	1.15 (0.64, 2.10)		1.13 (0.66, 1.93)		1.12 (0.69, 1.81)	
Endline	IG (*n* = 39)	3 (7.7)	36 (92.3)	2 (5.1)	37 (94.9)	12 (30.8)	27 (69.2)
	CG (*n* = 39)	14 (35.9)	25 (36.1)	11 (28.2)	28 (71.8)	15 (38.5)	24 (61.5)
	RR (95%CI)	0.21 (0.07, 0.69) *		0.18 (0.04, 0.77) *		0.80 (0.43, 1.48)	

SF = Skeletal Fluorosis (cannot do exercise); ^§^ Testing was done according to Shashi et al. [22]. * Statistically significant association observed at *p*-value < 0.05. IG = Intervention Group; CG = Control Group.

**Table 5:** Pain and muscular symptoms of non-skeletal fluorosis in a 6-month eggshell powder intervention between intervention and control groups.

		Pain and Muscular Symptoms ^§^ of Non-Skeletal Fluorosis, N (%)													
		Lower Back Pain		Leg Joints Pain		Arm JointPain		Neck Pain with Movement		Tingling, Hands and Feet		Muscle Weakness		Abdominal Pain	
	Group	Yes	No	Yes	No	Yes	No	Yes	No	Yes	No	Yes	No	Yes	No
**Baseline**	IG (*n* = 39)	30 (76.9)	9 (23.1)	27 (69.2)	12 (30.8)	21 (53.8)	18 (46.2)	20 (51.3)	19 (48.7)	27 (69.2)	12 (30.8)	25 (64.1)	14 (35.9)	22 (56.4)	17 (43.6)
	CG (*n* = 39)	26 (66.7)	13 (33.3)	23 (59.0)	16 (41.0)	16 (41.0)	23 (59.0)	22 (56.4)	17 (43.6)	28 (71.8)	11 (28.2)	24 (61.5)	15 (38.5)	24 (61.5)	15 (38.5)
	RR (95%CI)	1.15 (0.87, 2.53)		1.17 (0.84, 1.64)		1.31 (0.82, 2.11)		0.91 (0.60, 1.37)		0.96 (0.72, 1.28)		1.04 (0.74, 1.47)		0.92 (0.63, 1.33)	
**Endline**	IG (*n* = 39)	17 (43.6)	22 (56.4)	9 (23.1)	30 (76.9)	5 (12.8)	34 (87.2)	3 (7.7)	36 (92.3)	5 (12.8)	34 (87.2)	8 (20.5)	31 (79.5)	8 (20.5)	31 (79.5)
	CG (*n* = 39)	29 (74.4)	10 (25.6)	27 (69.2)	12 (30.8)	21 (53.8)	18 (46.2)	18 (46.2)	21 (53.8)	20 (51.3)	19 (48.7)	26 (66.7)	13 (33.3)	27 (69.2)	12 (30.8)
	RR (95%CI)	0.59 (0.39, 0.88) *		0.33 (0.18, 0.61) *		0.24 (0.10, 0.57) *		0.17 (0.05, 0.52) *		0.25 (0.10, 0.60) *		0.31 (0.16, 0.59) *		0.30 (0.15, 0.57) *	

^§^ Testing was done according to Shashi et al. [22]; * Statistically significant association observed at *p*-value < 0.05. IG = Intervention group, CG = Control group.

**Table 6:** Gastrointestinal and urinary symptoms of non-skeletal fluorosis in a 6-month eggshell powder intervention in treatment and control groups.

		Gastrointestinal and Urinary Symptoms ^§^ of Non-Skeletal Fluorosis, N (%)											
		Loss of Appetite		Nausea		Bloating in Stomach		Polydipsia		Polyuria		Constipation	
	Group	Yes	No	Yes	No	Yes	No	Yes	No	Yes	No	Yes	No
**Baseline**	IG (*n* = 39)	14 (35.9)	25 (64.1)	21 (53.8)	18 (46.2)	7 (17.9)	32 (82.1)	6 (15.4)	33 (84.6)	2 (5.1)	37 (94.9)	31 (79.5)	8 (20.5)
	CG (*n* = 39)	12 (30.8)	27 (69.2)	19 (48.7)	20 (51.3)	8 (20.5)	31 (79.5)	3 (7.7)	36 (92.3)	3 (7.7)	36 (92.3)	34 (87.2)	5 (12.8)
	RR (95%CI)	1.15 (0.87, 2.53)		1.17 (0.84, 1.64)		1.31 (0.82, 2.11)		0.91 (0.60, 1.37)		0.96 (0.72, 1.28)		1.04 (0.74, 1.47)	
**Endline**	IG (*n* = 39)	4 (10.3)	35 (89.7)	5 (12.8)	34 (87.2)	7 (17.9)	32 (82.1)	5 (12.8)	34 (87.2)	2 (5.1)	37 (94.9)	10 (25.6)	29 (74.4)
	CG (*n* = 39)	17 (43.6)	22 (56.4)	21 (53.8)	18 (46.2)	9 (23.1)	30 (76.9)	7 (17.9)	32 (82.1)	4 (10.3)	35 (89.7)	13 (33.3)	26 (66.7)
	RR (95%CI)	0.23 (0.09, 0.64) *		0.24 (0.10, 0.58) *		0.78 (0.32, 1.88)		0.71 (0.25, 2.06)		0.50 (0.10, 2.57)		0.77 (0.38, 1.54)	

^§^ Testing was done according to Shashi et al. [22]; * Statistically significant association observed at *p*-value < 0.05. IG = Intervention group, CG = Control group.

## Data Availability

Data will be made available upon request.
